# Supplementary material for: Protein conformational transitions explored by a morphing approach based on normal mode analysis in internal coordinates
Source: PLoS One. 2021 Nov 4;16(11):e0258818. doi: 10.1371/journal.pone.0258818 (PMC8568156; doi:10.1371/journal.pone.0258818)
Supplement: S1 Text — (DOCX) [file pone.0258818.s001.docx]

**S1 Text**

**The ICONGENI simulations to explore ADK transition mechanisms via the NMP_O_ state**

Although the transition mechanisms of ADK, well known in previous studies [1–9], are divided into two scenarios: the transition via the NMP-closing/LID-opening (NMP_C_) state and that via the NMP-opening/LID-closing (NMP_O_) state, the pathway ensemble generated by ICONGENI supports only the existence of the NMP_C_ state (**Fig 7**). This simulation implies that the flexibility of the interdomain between NMP and CORE is higher than that between LID and CORE, resulting from that the vibrational features describing variations in the NMP-CORE angle are arranged in the lowest normal mode.

However, it does not mean ICONGENI always returns a single candidate of transition paths. To demonstrate that ICONGENI can explore multiple transition pathways, we performed extra simulations where the paths via the NMP_O_ state not detected in the pathway ensemble simulated by ICONGENI were explored. The strategy was to use the normal mode set, composed of lowest normal modes characterizing the dynamics of $\theta_{LID}$ rather than those of $\theta_{NMP}$, as the DOFs of the simulations. To determine if each normal mode has the desired mode shape, the changes of $\theta_{NMP}$ and $\theta_{LID}$ on the $k^{th}$ normal mode shape ($\Delta\theta_{NMP,k}$ and $\Delta\theta_{LID,k}$) were measured. In detail, if $\boldsymbol{X}$ is a target intermediate structure in which the NMA calculation is performed, the structure deformed by the $k^{th}$ normal mode, $\boldsymbol{X'}_{k}$, is defined as

$\boldsymbol{X'}_{k}\boldsymbol{=}\boldsymbol{X}_{i}+50\times\Delta\boldsymbol{v}_{k}$, (1)

where $\Delta\boldsymbol{v}_{k}$ is the normalized eigenvector of the $k^{th}$ normal mode.

Then, $\Delta\theta_{NMP(or LID),k}$ is determined as follows.

$\Delta\theta_{NMP(or LID),k}=\left| \theta_{NMP(or LID)}\left( \boldsymbol{X} \right)-\theta_{NMP(or LID)}\left( \boldsymbol{X'}_{k} \right) \right|$, (2)

where $\theta_{NMP(or LID)}\left( \boldsymbol{S} \right)$ is the value of $\theta_{NMP(or LID)}$ of the structure $\boldsymbol{S}$.

If $\Delta\theta_{LID,k}-\Delta\theta_{NMP,k}>a$ ($a$ is a positive threshold value), the $k^{th}$ mode is judged to have the vibrational characteristics where interdomain motion between LID and CORE is more dominant than that between NMP and CORE.

In this simulation work, we performed the ICONGENI simulations by changing the value of $a$ (= 3, 3.5, 4, 4.5, 5, 5.5, and 6 degrees) in Eq. (2) and confirmed that ICONGNEI successfully explored the transition pathways via the NMP­_O_ state (**S1 Fig**). In conclusion, our technique can predict multiple transition pathways compatible to several metastable states if their structural information is given.

**References**

1. Berry MB, Phillips Jr GN. Crystal structures of Bacillus stearothermophilus adenylate kinase with bound Ap5A, Mg2+ Ap5A, and Mn2+ Ap5A reveal an intermediate lid position and six coordinate octahedral geometry for bound Mg2+ and Mn2+. Proteins. 1998; 32(3):276–288. https://doi.org/10.1002/(SICI)1097-0134(19980815)32:3<276::AID-PROT3>3.0.CO;2-G PMID: 9715904
2. Schlauderer GJ, Proba K, Schulz GE. Structure of a mutant adenylate kinase ligated with an ATP-analogue showing domain closure over ATP. J Mol Biol. 1996; 256(2):223–227. https://doi.org/10.1006/jmbi.1996.0080 PMID: 8594191
3. Maragakis P, Karplus M. Large amplitude conformational change in proteins explored with a plastic network model: adenylate kinase. J Mol Biol. 2005; 352(4):807–822. https://doi.org/10.1016/j.jmb.2005.07.031 PMID: 16139299
4. Jana B, Adkar BV, Biswas R, Bagchi B. Dynamic coupling between the LID and NMP domain motions in the catalytic conversion of ATP and AMP to ADP by adenylate kinase. J Chem Phys. 2011; 134(3):035101. https://doi.org/10.1063/1.3516588 PMID: 21261390
5. Wang Y, Gan L, Wang E, Wang J. Exploring the dynamic functional landscape of adenylate kinase modulated by substrates. J Chem Theory Comput. 2013; 9(1):84–95. https://doi.org/10.1021/ct300720s PMID: 26589012
6. Lin CY, Huang JY, Lo LW. Deciphering the catalysis-associated conformational changes of human adenylate kinase 1 with single-molecule spectroscopy. J Phys Chem B. 2013; 117(45):13947–13955. https://doi.org/10.1021/jp4019537 PMID: 24134437
7. Kong J, Li J, Lu J, Li W, Wang W. Role of substrate-product frustration on enzyme functional dynamics. Phys Rev E. 2019; 100(5):052409. https://doi.org/10.1103/PhysRevE.100.052409 PMID: 31869999
8. Beckstein O, Denning EJ, Perilla JR, Woolf TB. Zipping and unzipping of adenylate kinase: atomistic insights into the ensemble of open↔ closed transitions. J Mol Biol. 2009; 394(1):160–176. https://doi.org/10.1016/j.jmb.2009.09.009 PMID: 19751742
9. Oshima H, Re S, Sugita Y. Replica-Exchange Umbrella Sampling Combined with Gaussian Accelerated Molecular Dynamics for Free-Energy Calculation of Biomolecules. J Chem Theory Comput. 2019; 15(10):5199–5208. https://doi.org/10.1021/acs.jctc.9b00761 PMID: 31539245
