# Supplementary figures and images for: Protein conformational transitions explored by a morphing approach based on normal mode analysis in internal coordinates
Source: PLoS One. 2021 Nov 4;16(11):e0258818. doi: 10.1371/journal.pone.0258818 (PMC8568156; doi:10.1371/journal.pone.0258818)

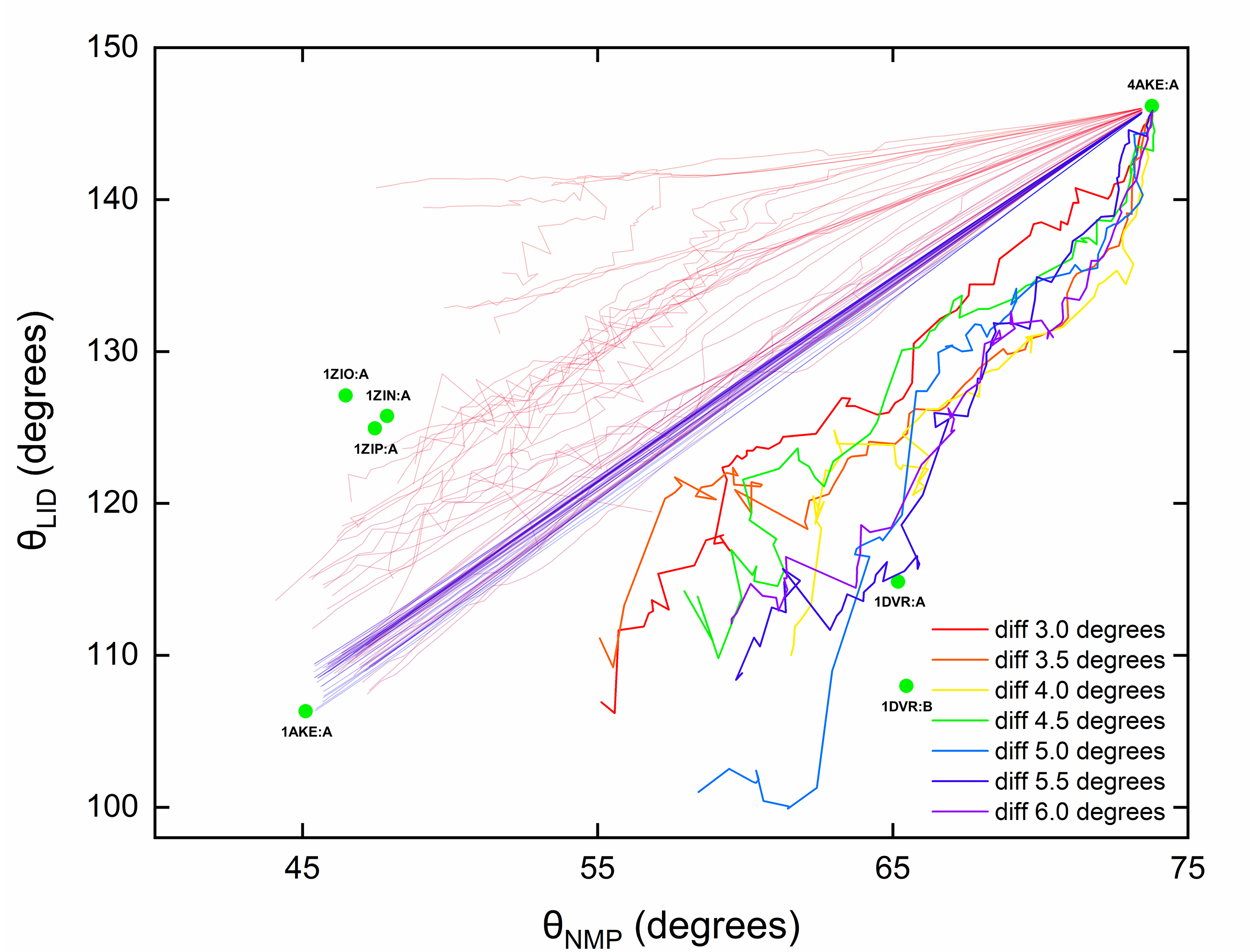

Supplement: S1 Fig — Seven pathways in the vicinity of the NMPO state are shown on θNMP−θLID space. The pathway named “diff. a degrees” means that it was explored by ICONGENI using the lowest normal modes that satisfy the condition: ΔθLID−ΔθNMP>a (see details in S1 Text). The ADK crystal structures are taken as the references (indicated by green circles). 4AKE:A and 1AKE:A indicate the open and closed states of ADK, respectively. 1ZIN:A, 1ZIO:A, and 1ZIP:A (1DVR:A, and 1DVR:B) indicate experimental structures at the NMPC state (the NMPO state). The pathway ensemble data (Fig 7B) is also included in this figure for comparison. (TIF) [file pone.0258818.s002.tif]
