# Supplementary material for: Protein conformational transitions explored by a morphing approach based on normal mode analysis in internal coordinates
Source: PLoS One. 2021 Nov 4;16(11):e0258818. doi: 10.1371/journal.pone.0258818 (PMC8568156; doi:10.1371/journal.pone.0258818)
Supplement: Attachment — Submitted filename: Response to Reviewers.docx [file pone.0258818.s005.docx]

**Rebuttal**

**Manuscript Title:** Protein Conformational Transitions Explored by a Morphing Approach Based on Normal Mode Analysis in Internal Coordinates

**Authors:** Byung Ho Lee, Soon Woo Park, Soojin Jo, Moon Ki Kim

First of all, we thank the referees for providing thoughtful and helpful suggestions and comments. In the revised manuscript, we have incorporated changes motivated by them. In what follows, the referee’s comments are listed, with our responses interspersed in blue. All responses begin with “Response of the Authors:”

**Reviewers’ comments:**

**Reviewer #1:** The authors proposed an interesting path-sampling method to when end-point structures are given a priori. This method was developed based on a morphing algorithm and the normal mode analysis (NMA) and allows us to generate intermediate states between the end-point structures with low computational costs. The theoretical part was organized well by referring to their previous studies for this extension study. However, there might be some issues to be addressed prior to actual applications. Therefore, I would like to ask the following issues before publishing.

1) This method provides a set of (or an ensemble) of transition paths between given end-point structures. However, it is difficult to directly calculate free energy profiles based on this method when evaluating generated paths. In actual applications, users want to evaluate generated transition paths quantitatively on their free energy profiles. Herein, how to evaluate the generated paths quantitatively using some physical quantities? Are there additional treatments for the quantitative evaluations? I would like to know a way to validate the generated transition paths.

**Response of the Authors:** In our manuscript, we performed a comparative analysis of NGENI and ICONGENI for two protein cases (ADK and RBP) and investigated the distributions of backbone bond length and bond angle terms on the corresponding pathways to verify the superiority of ICONGENI in terms of chemical stability. According to referee’s suggestion, we performed a further quantitative analysis to evaluate these two methods. In the same context as analyzing the variations of the internal coordinates (*i.e.*, backbone bond length and bond angle), we calculated the potential energy of the intermediate conformations on the pathways. Because both simulation methods were carried out by using a coarse-grained modeling method, non-backbone atoms and O atom in the backbone from reference structures (4AKE:A and 1BA2:A for ADK and RBP, respectively) were grafted to all intermediates and the generated all atom models were energy minimized within CHARMM36m force field [1] to eliminate any steric clashes and inappropriate geometries. Next, we calculated the potential energies of all intermediates of the NGENI and ICONGENI pathways by using CHARMM36m force field to quantitatively evaluate how the corresponding transitions are stable. **Fig R1** shows the difference of the potential energy of each frame between the NGENI and ICONGENI pathways. From the results, we confirm that the ICONGENI pathways are generally more stable than the NGENI pathways. Given that the geometric errors are gradually accumulated when anharmonic transitions are explored by harmonic modes, their energy gap increases as the pathways progress. These simulation results imply that the mode shapes in internal coordinates are more suitable for exploring large-scale transitions than those in Cartesian coordinates. In the revised manuscript, we clarify this point by adding the following sentences on Page 21 and **Fig R1** as **Fig 6**, with the following captions.

In the same context as investigating the bond length and bond angle distributions, the potential energies of the intermediate conformations comprising the resulting pathways were calculated. Because both simulation methods were carried out by using a coarse-grained modeling method, non-backbone atoms and O atom in the backbone from reference structures (4AKE:A and 1BA2:A for ADK and RBP, respectively) were grafted to all intermediates and the generated all atom models were energy minimized within CHARMM36m force field for 500 steps of conjugate gradient [37] to eliminate any steric clashes and inappropriate geometries. Next, we calculated the potential energies of all intermediates of the NGENI and ICONGENI pathways by using CHARMM36m force field to quantitatively evaluate how the corresponding transitions are stable. **Fig 6** shows the difference of the potential energy of each frame between the NGENI and ICONGENI pathways. From the results, we confirmed that the ICONGENI pathways are generally more stable (*i.e.*, having lower potential energies) than the NGENI pathways. Furthermore, their energy gap increased as the pathways progress, which suggests that the qualitative difference between the pathways is increasingly noticeable in that the geometric errors are gradually accumulated when anharmonic transitions are explored by harmonic modes. Finally, these simulation results imply that the ICONGNIE pathways are more reliable than the NGENI pathways in terms of thermal and chemical stability.

**Fig 6.** **The difference between potential energies of ICONGENI and NGENI pathways for (A) ADK and (B) RBP.** The difference of the potential energy $\Delta U=U_{ICONGENI}-U_{NGENI}$. Before calculating the potential energies, all simulated intermediate structures were transformed into all atom models based on corresponding reference structures (4AKE:A (1BA2:A) for ADK (RBP)) and were energy minimized using CHARMM36m force field for 500 steps of conjugate gradient.

**Fig R1. The difference between the potential energies of the ICONGENI and NGENI pathways for (A) ADK and (B) RBP.** The difference of the potential energy $\Delta U=U_{ICONGENI}-U_{NGENI}$. Before calculating the potential energies, all simulated intermediate structures were transformed into all atom models based on corresponding reference structures (4AKE:A (1BA2:A) for ADK (RBP)) and were energy minimized using CHARMM36m force field for 500 steps of conjugate gradient.

2) In this study, the authors specified the 50 lowest modes as DOFs for the linear combinations. Why did you specify these modes? Are there any conditions to specify optimal DOFs to generate reliable transition paths? I think that users would like to know a way to specify the optimal DOFs.

**Response of the Authors:** In the original manuscript, we considered 50 lowest normal modes as an enough number of DOFs to explore the transition pathways of ADK and RBP in terms of pathway convergence. According to PDB statistics, most of proteins (more than 93 percent of all structures solved by X-ray crystallography or electron microscopy) has lower than 1.4 Å resolution. Therefore, we measured the root-mean-square deviations (RMSDs) of the consecutive intermediates comprising the paths with respect to the final conformation (*i.e.*, the closed structures) and judged that the corresponding paths meet the convergence condition if the RMSD values steadily decrease below 1.4 Å. **Table R1** shows the minimum values of RMSD of each pathway depending on the number of lowest normal modes used in the simulation for the two proteins. From the results, we determined that 50 is the minimum and optimal number of the modes to satisfy the convergence condition in both cases. In the revised manuscript, we add the following sentence on Page 17 for better clarification.

The DOFs of these simulations were set to be the 50 lowest normal modes considered empirically sufficient to simulate the conformational changes within the experimental resolution based on our previous study [25].

**Table R1. Evaluation of pathway convergence of ICONGENI for two proteins**

|  | **ADK** | **RBP** |
| --- | --- | --- |
| **# of lowest normal modes** | **RMSD (Å)**^a^ | **RMSD (Å)** |
| 10 | 3.20 | 3.22 |
| 20 | 2.26 | 3.21 |
| 30 | 1.72 | 2.76 |
| 40 | 1.26 | 1.72 |
| 50 | 1.08 | 1.32 |
| 60 | 1.02 | 1.16 |
| 70 | 0.94 | 0.91 |
| 80 | 0.90 | 0.65 |
| 90 | 0.81 | 0.53 |
| 100 | 0.78 | 0.52 |

Note that gray shading indicates the simulation cases where the convergence condition is not satisfied.

^a^ The minimum value of RMSD of the corresponding path with respect to the final reference structure.

3) Does this method work if there are multiple transition paths between given end-point structures? Does the current strategy return one of the multiple transition paths? Does this method always return a single candidate of transition paths? Or can this method always return the minimum free energy path (MFEP)?

**Response of the Authors:** Fundamentally, ICONGENI generates a deterministic pathway between given end-point structures under a specific condition (*e.g.*, a set of the lowest normal modes used in simulation and the number of iteration steps). In the original manuscript, we tried to generate the multiple transition paths for ADK depending on the number of the lowest normal modes used in ICONGENI simulation (**Fig 6**). Although the transition mechanisms of ADK, well known in previous studies [2], are divided into two scenarios: the transition via the NMP-closing/LID-opening (NMP_C_) state and that via the NMP-opening/LID-closing (NMP_O_) state, the pathway ensemble generated by ICONGENI supports only the existence of the NMP_C_ state (**Fig 6**). This simulation implies that the flexibility of the interdomain between NMP and CORE is higher than that between LID and CORE, resulting from that the vibrational features describing variations in the NMP-CORE angle are arranged in the lowest normal mode.

However, it does not mean ICONGENI always returns a single candidate of transition paths. To demonstrate that ICONGENI can explore multiple transition pathways, we performed extra simulations where the paths via the NMP_O_ state not detected in the pathway ensemble simulated by ICONGENI were explored. The strategy was to use the normal mode set, composed of lowest normal modes characterizing the dynamics of $\theta_{LID}$ rather than those of $\theta_{NMP}$, as the DOFs of the simulations. As a result, ICONGNEI successfully generated the transition pathways via the NMP­_O_ state (**Fig R2**), which implies that if information of metastable states of a protein is given, our technique can explore the transition pathways compatible to the given states. In the revised manuscript, we add following sentences to discuss this point.

However, this result does not mean ICONGENI always returns a single candidate (*i.e.*, paths via the NMP_C_ state) of the transition paths. If the normal mode set as the system DOFs is determined under certain conditions, ICONGENI could explore transition pathways via the NMP_O_ state, which demonstrate that ICONGENI can explore multiple transition pathways compatible to several metastable states if information of the states is given (See more details in **S1 Text** and **S1 Fig**).

Furthermore, this discussion is included in the supporting information **S1 Text**. **Fig R2** is also included as **S1 Fig** with following captions.

**S1 Fig.** Projection of the ICONGENI pathways for ADK onto $\theta_{NMP}$–$\theta_{LID}$ space. Six pathways in the vicinity of the NMP_O_ state are shown on $\theta_{NMP}$–$\theta_{LID}$ space. The pathway named “diff. $a$ degrees” means that it was explored by ICONGENI using the lowest normal modes that satisfy the condition: $\Delta\theta_{LID}- \Delta\theta_{NMP}>a$ (see details in **S1 Text**). The ADK crystal structures are taken as the references (indicated by green circles). 4AKE:A and 1AKE:A indicate the open and closed states of ADK, respectively. 1ZIN:A, 1ZIO:A, and 1ZIP:A (1DVR:A, and 1DVR:B) indicate experimental structures at the NMP_C_ state (the NMP_O_ state). The pathway ensemble explored by ICONGENI is also shown on $\theta_{NMP}$–$\theta_{LID}$ space for comparison (The image in **Fig 6B** is blurred).

**Fig R2. Projection of the ICONGENI pathways for ADK onto** $\boldsymbol{\theta}_{\boldsymbol{NMP}}$**–**$\boldsymbol{\theta}_{\boldsymbol{LID}}$ **space.** Six pathways in the vicinity of the NMP_O_ state are shown on $\theta_{NMP}$–$\theta_{LID}$ space. To explore the NMP_O_ state, the specific normal modes that characterize the dynamics of $\theta_{LID}$ are used to generate the transition pathways. To select the desired normal modes, the changes of $\theta_{NMP}$ and $\theta_{LID}$ in the $k^{th}$ normal mode ($\left( \Delta\theta_{NMP} \right)_{k}$ and $\left( \Delta\theta_{LID} \right)_{k}$) are measured by the following equation: $\left( \Delta\theta_{NMP(or LID)} \right)_{k}=\left| \theta_{NMP(or LID)}\left( \boldsymbol{X} \right)-\theta_{NMP(or LID)}\left( \boldsymbol{X}_{deform,k} \right) \right|$, where $\boldsymbol{X}_{i}$ is the position vectors of atom $i$ of the intermediate structure and $\left( \boldsymbol{X}_{deform,k} \right)_{i}\boldsymbol{=}\boldsymbol{X}_{i}+50\times\Delta\boldsymbol{r}_{i,k}$ ($\Delta\boldsymbol{r}_{i,k}$ is the normalized eigenvector of atom $i$ in the $k^{th}$ normal mode). The pathway named “diff. $a$ degrees” means that ICONGENI uses the normal modes that satisfy the condition: $\Delta\theta_{LID}- \Delta\theta_{NMP}>a$. The ADK crystal structures are taken as the references (indicated by green circles). 4AKE:A and 1AKE:A indicate the open and closed states of ADK, respectively. 1ZIN:A, 1ZIO:A, and 1ZIP:A (1DVR:A, and 1DVR:B) indicate experimental structures at the NMP_C_ state (the NMP_O_ state). The existing pathway ensemble depending on the number of lowest normal modes is also shown on $\theta_{NMP}$–$\theta_{LID}$ space for comparison (The image in **Fig 6B** is blurred).

Lastly, we cannot guarantee that ICONGENI produces the minimum free energy path, but we could say that ICONGENI is able to generate transition pathways that minimize a pseudo-energy cost function (*i.e.*, a cost function is defined as a similar form of potential energy). In our manuscript, the evaluation of whether the ICONGENI pathways for ADK are effective and reliable in terms of free energy was also performed by using its interdomain angle space that indirectly represents free energy landscapes.

4) For Eq (23), a target intermediate is determined by the linear interpolation between given end-point structures. Is this assumption enough to describe complicated transition paths of proteins? My concern is that this simple linear interpolation might include some artifact when we must consider complicated transitions of proteins between given end-point structures.

**Response of the Authors:** As the referee pointed out, it is difficult for our method to predict complicated transitions sensitive to a folding order. For example, the tRNA molecule has a distinctive folded structure that depends on the order of folding, so it is hard to predict valid pathways through simple interpolation technique between two end-point structures without any extra information on the intermediate conformations. Even in this case, if the structural information of the metastable states is available, our method can explore the pathways through independent simulations at each folding stage.

5) Related to previous issue (the 3rd issue), did this method successfully detect multiple transition paths of AK? In previous studies, several enhanced sampling methods detected multiple transitions path between the open and closed states of AK (for example, J. Chem. Theory Comput, 2019, 15, 5199-5208, **Fig 5**). I would like to a correspondence of your result with previous study’s results.

**Response of the Authors:** In the original manuscript, we briefly introduced the metastable states (including the NMP_C_ state and the NMP_O_ state) that several theoretical and experimental studies revealed [3­­–10], and in question 3, it was confirmed our method successfully explored multiple transition paths (*i.e.*, via the NMP_C_ state and via the NMP_O_ state).

Given that ADK has multiple pathways along the open/close transitions and with or without ligands, various results of ADK transitions can be produced depending on technical frameworks. For example, GaReus (proposed in J. Chem. Theory Comput, 2019, 15, 5199-5208) generated the free energy landscapes where the NMP_O_ state appears clearly and the NMP_C_ state does not. On the other hand, our method explored the ADK transitions based on the dynamic characteristics of ADK without ligand binding and provided supporting evidence that those via the NMP_C_ state is more favorable than those via the NMP_O_ state. In the revised manuscript, the article mentioned above is cited.

6) Why did you adopt the ENM potential as a coarse-grained (CG) potential? Are there any possibilities to specify the other CG potentials? Generally, I think that the ENM potential is used to describe harmonic modes around a relaxed configurations using a reference structure such as an experimental (X-ray crystal or NMR) structure. Therefore, I think that this ENM potential might be unsuitable for describing large-amplitude domain motions. Why can this ENM potential treat or induce anharmonic motions of AK or RBP?
**Response of the Authors:** My research interests lie in developing computational methods based on ENM potential to analyze conformational transitions of proteins. As the referee pointed out, conformational transition events are generally described by anharmonic motions, and there are limitations to predict the exact pathways of the events. Therefore, in this manuscript, we proposed a novel computational approach to predict the paths by bridging the gap between the anharmonic dynamics and the harmonic dynamics (*i.e.*, lowest mode shapes).

7) I think there might be a typo on Eq (17). It seems that the chain rule on Eq. (17) does not work correctly in the present description.

**Response of the Authors:** In the revised manuscript, this typo is fixed.

**References**

1. Huang J, Rauscher S, Nawrocki G, Ran T, Feig M, De Groot BL, et al. CHARMM36m: an improved force field for folded and intrinsically disordered proteins. Nat Methods. 2017; 14(1):71–73. https://doi.org/10.1038/nmeth.4067 PMID: 27819658
2. Wang Y, Gan L, Wang E, Wang J. Exploring the dynamic functional landscape of adenylate kinase modulated by substrates. J Chem Theory Comput. 2013; 9(1):84–95. https://doi.org/10.1021/ct300720s PMID: 26589012
3. Berry MB, Phillips Jr GN. Crystal structures of Bacillus stearothermophilus adenylate kinase with bound Ap5A, Mg2+ Ap5A, and Mn2+ Ap5A reveal an intermediate lid position and six coordinate octahedral geometry for bound Mg2+ and Mn2+. Proteins. 1998; 32(3):276–288. https://doi.org/10.1002/(SICI)1097-0134(19980815)32:3<276::AID-PROT3>3.0.CO;2-G PMID: 9715904
4. Schlauderer GJ, Proba K, Schulz GE. Structure of a mutant adenylate kinase ligated with an ATP-analogue showing domain closure over ATP. J Mol Biol. 1996; 256(2):223–227. https://doi.org/10.1006/jmbi.1996.0080 PMID: 8594191
5. Maragakis P, Karplus M. Large amplitude conformational change in proteins explored with a plastic network model: adenylate kinase. J Mol Biol. 2005; 352(4):807–822. https://doi.org/10.1016/j.jmb.2005.07.031 PMID: 16139299
6. Jana B, Adkar BV, Biswas R, Bagchi B. Dynamic coupling between the LID and NMP domain motions in the catalytic conversion of ATP and AMP to ADP by adenylate kinase. J Chem Phys. 2011; 134(3):035101. https://doi.org/10.1063/1.3516588 PMID: 21261390
7. Wang Y, Gan L, Wang E, Wang J. Exploring the dynamic functional landscape of adenylate kinase modulated by substrates. J Chem Theory Comput. 2013; 9(1):84–95. https://doi.org/10.1021/ct300720s PMID: 26589012
8. Lin CY, Huang JY, Lo LW. Deciphering the catalysis-associated conformational changes of human adenylate kinase 1 with single-molecule spectroscopy. J Phys Chem B. 2013; 117(45):13947–13955. https://doi.org/10.1021/jp4019537 PMID: 24134437
9. Kong J, Li J, Lu J, Li W, Wang W. Role of substrate-product frustration on enzyme functional dynamics. Phys Rev E. 2019; 100(5):052409. https://doi.org/10.1103/PhysRevE.100.052409 PMID: 31869999
10. Beckstein O, Denning EJ, Perilla JR, Woolf TB. Zipping and unzipping of adenylate kinase: atomistic insights into the ensemble of open↔ closed transitions. J Mol Biol. 2009; 394(1):160–176. https://doi.org/10.1016/j.jmb.2009.09.009 PMID: 19751742

**Reviewer #2:** Comments for the Authors

Lumping protein structures are undergoing large-scale conformational changes, which are essential to associate with their function at the cell and organism level, but have been elusive both experimentally and computationally. In this study, Lee et al. proposed a new computational methodology called internal coordinate normal mode-guided elastic network interpolation (ICONGENI) to sample intermediate conformations by interpolating the interatomic distance between two end-point conformations with the degrees of freedom constrained by the low-frequency dynamics afforded by normal mode analysis (NMA) in internal coordinates. The applications to two case studies (adenylate kinase (ADK) and ribose-binding protein (RBP)) by analyzing their open-to-closed transitions show that ICONGENI can explore highly reliable pathways in terms of chemical and thermal stability. Take together, the method illustrated in this study exhibits a certain degree of potentiality in terms of intermediated conformations predictions for these proteins with large-scale conformational changes during functioning. This study is a continuous work to the previous one, basically by changing the position descriptor of cartesian coordinates to that of internal coordinates. However, it’s superiority was not emphasized enough by the way of presenting/writing in this manuscript.

There are several points described below,

1. What are the computational costs for ICONGENI and NGENI methods? Is ICONGENI method much more computationally cheaper than NGENI?

**Response of the Authors:** The distinct difference between ICONGENI and NGENI is the type of the coordinate system where NMA calculation is performed: IC-NMA and CC-NMA in ICONGENI and in NGENI, respectively. Given that IC-NMA has extra calculations involved in transformation either from CCs to ICs or from ICs to CCs, ICONGENI requires higher computational complexity than does NGENI. However, ICONGENI can provide chemically relevant mode shapes preserving the secondary structure of proteins and extending the validity of the harmonic approximation required for the calculation in that internal coordinates (ICs) define molecular geometry using bond length, bond angle, and torsion angle terms [1–3]. In the revised manuscript, we clarify this point by adding the following sentences on Page 4.

In this study, we propose a new NMA-based pathway generation method called internal coordinate normal mode-guided elastic network interpolation (ICONGENI), an improved technique over the normal mode-guided elastic network interpolation (NGENI) [25]. The fundamental concept of both methods is to obtain intermediate conformations comprising a transition pathway by iteratively calculating displacement vectors to minimize error between the initial structure and the desired intermediates. The critical difference between the two techniques is in NMA calculation performed to represent the displacement vectors as linear combinations of the lowest normal mode shapes: NMA calculation is achieved in internal coordinates (IC-NMA) and Cartesian coordinates (CC-NMA) in ICONGENI and NGENI, respectively. CC-NMA has been widely used in studying protein dynamics due to the inherent nature of Cartesian coordinates (CCs): high computational efficiency and intuitive expression of protein dynamics but has a critical weakness that the resulting mode shapes unavoidably have unrealistic distortions like bond length stretching and bond angle bending [26]. On the other hand, IC-NMA has a distinctive advantage in describing the large-scale transitions. It has been well known that large-scale transitions of proteins are dominantly influenced by the variations in the torsion angles, not in the bond lengths and the bond angles that are nearly rigid. Internal coordinates (ICs) facilitate to separate the torsion angle terms from the others, so that IC-NMA can be performed in torsion angle space. This strategy enables it to produce chemically relevant mode shapes preventing the unrealistic distortions of bond lengths and bond angles and extending the validity of the harmonic approximation in calculation [26–28]. In terms of computational complexity, IC-NMA is less efficient than CC-NMA because it has extra calculations involved in transformation either from CCs to ICs or from ICs to CCs (see section 2.2 for further details), but this is not a critical point in that both methods can be performed at the personal computer level. In other words, IC-NMA is more suitable than CC-NMA for describing and exploring large-scale conformational changes of proteins, which provides an insight into the development of ICONGENI.

2. The energy minimization details should be provided. Such as what is the minimization method, how many steps.

**Response of the Authors:** According to referee’s suggestion, we provided the energy minimization details in Page 6 of the revised manuscript.

The selected structures were refined by energy minimization (500 steps of conjugate gradient) using the CHARMM36m force field [37].

3. What is the cutoff distance used in the elastic network model (ENM)? What is the correlation between this parameter and the predicted results?

**Response of the Authors:** Thanks to the referee’s review, we recognized that we omitted information about cutoff distance in the original manuscript. As the bottom line, the cutoff distance was set to 12 Å in our study because we empirically verified that the optimal value of the cutoff distance is 12 Å in the ENM [4]. In detail, the conventional ENM simplifies all-atom structures into mass-spring networks where only alpha carbons are sampled and linked each other by linear springs. In the coarse-grained model, the linear springs should connect up to four consecutive alpha carbons to normally represent the dynamic behavior of proteins (*i.e.*, variations of bond length, bond angle, and torsion angle). Therefore, 12 Å could be a minimum threshold in that the interatomic distance of two consecutive alpha carbons is about 3.8 Å [5].

When the cutoff distance is set to be greater than or less than 12 Å, NMA calculation has some issues. Large cutoff distance increases the number of interatomic connections, leading to that the computational cost increases exponentially. On the other hand, short cutoff distance causes more than six zero eigenvalues corresponding to rigid body motions in NMA calculation because a system could be underdetermined in this loose network model, leading to the unrealistic dynamic behaviors of the system. Therefore, we decided to use 12 Å as the cutoff distance in this manuscript. In the revised manuscript, we added the following sentence is added on Page 8 to provide information about cutoff distance.

$d_{cutoff}$ was set to be 12 Å [19].

4. “For both proteins, the avg (std) values in the ICONGENI pathways were more concentrated around (below) the experimental values than were those in the NGENI pathways (**Fig 4**).” Here, the authors should provide more elaborate discussion regarding the reason behind this distribution difference.

**Response of the Authors:** According to referee’s suggestion, we provided further discussion on the superiority of ICONGENI over NGENI in terms of chemical stability as the following sentences on Page 18 in the revised manuscript.

Next, we investigated backbone bond length and bond angle distributions on the simulated pathways. Proteins undergo conformational changes mainly through variations of two types of backbone torsion angles: phi ($\phi$) around the $N-C_{\alpha}$ bond and psi ($\psi$) around the $C_{\alpha}-C$ bond while variations of backbone bond lengths and bond angles are impractical during the transitions. The distributions of the bond length and bond angle provides key information to evaluate how well the proteins keep their molecular shape during large deformation. First, the backbone bond lengths are divided into three types: $N-C_{\alpha}$, $C_{\alpha}-C$, and $C-N$. According to the type, we calculated average (avg) and standard deviation (std) values over ICONGENI and NGENI pathways for two proteins (ADK and RBP) and analyzed their distributions using the experimental data as the reference avg and std values of the backbone bond length types (**Fig 4**) [45]. The avg values of the bond length in the ICONGENI paths were more concentrated around the corresponding experimental values than were those in the NGENI paths. Likewise, most std values of the bond length terms in the ICONGENI paths were distributed below the corresponding experimental values while many of those in the NGENI paths were higher than the experimental values. Subsequently, we investigated the backbone bond angle distributions (including $N-C_{\alpha}-C$, $C_{\alpha}-C-N$, and $C-N-C_{\alpha}$) of the simulated pathways in the same manner as described above for analysis of the bond length distributions. Similar to the results in the bond length distribution graphs, the avg values of the bond angles in the ICONGENI paths were densely distributed around the corresponding experimental values, and the std values were distributed close to zero compared to the NGENI pathways (**Fig 5**). These quantitative data commonly showed that the ICONGENI pathways were more likely to prevent unrealistic distortions in bond lengths and bond angles than were the NGENI pathways.

5. “While the simulation result for RBP showed that ICONGENI suffered more from the tip effect than did NGENI, this was not a critical issue because the distortions of the tip parts could be considered local vibrations that barely contributed to the conformational transitions” Why did ICONGENI suffer more from the tip effect than did NGENI? Is this a limitation for this method potentially?

**Response of the Authors:** We thank the referee for his/her thorough review which gave us another chance to deeply discuss the tip effect. In the original manuscript, we confirmed that the unrealistic distortions of bond lengths in ICONGENI pathways (denoted by the pink circles in **Fig 4B**) were in either the first or last residue and explained this result with the tip effect. The tip effect, as an inherent weakness of NMA, describes that some lowest normal modes have the unrealistic behavior of the highly flexible parts in protein structures (*e.g.*, hanging loops and protruding ends) [6]. NMA in internal coordinates (IC-NMA) is performed considering only backbone torsion angle dynamics, while the backbone bond lengths and bond angles are nearly fixed. Given that a torsion angle is defined by four consecutive atoms, the resulting mode shapes in IC-NMA is intrinsically limited in describing the atomic variations in the first or last residue where any torsion angle cannot be defined. Therefore, we concluded that IC-NMA might be more vulnerable to the tip effect than NMA in Cartesian coordinates that specifies the position of each atom. However, this limitation is not a critical issue when exploring large-scale transition pathways because the tip effect can be regarded as local deformation that has little effect on the global motions. In the revised manuscript, we add the following sentences on Page 19 for better clarification.

On the other hand, there were exceptions to this principle, such as the small number of bond length terms of the ICONGENI path with irrational avg or std values that deviated farther from the corresponding experimental ones than did those of the NGENI path (denoted by pink circles in **Fig 4B**). We confirmed that all bond length terms that fell under these exceptions belonged to either the first or last residue of the proteins, which would imply that these unrealistic distortions were caused by the tip effect [41]. The tip effect is an inherent weakness of NMA (regardless of the type of coordinate system) in which the highly flexible parts in protein structures (*e.g.*, hanging loops and protruding ends) exhibit abnormal behavior in some of the lowest normal modes. IC-NMA may suffer more from the tip effect than did CC-NMA because the mode shapes in IC-NMA is intrinsically limited in describing the movements of either the first or last residue where any torsion angle cannot be defined, which can explain the exceptions in **Fig 4B**. However, this limitation of ICONGENI is not a critical issue in predicting the transition pathways because the distortions of the tip parts could be considered as local vibrations that has little effect on the global motions.

6. Are the structures of the intermediated states along the predicted pathways extractable? If so, their structures should be presented (if too many intermediated structures, the authors can make a movie). If not, why?

**Response of the Authors:** Of course, the intermediate conformations comprising the simulated pathways are extractable. According to referee’s suggestion, we make movies for ICONGENI and NGENI pathways of ADK and RBP. In the revised manuscript, we add the following sentence on Page 17 and the movie files in the supporting information **S1 movie** and **S2 movie** with the following captions.

For better understanding, the transition pathways explored by ICONGENI of ADK and RBP are provided in **S1 movie** and **S2 movie**, respectively.

**S1 Movie. The transition pathway of ADK simulated by ICONGENI based on the DOFs of the 50 lowest normal modes.**

**S2 Movie. The transition pathway of RBP simulated by ICONGENI based on the DOFs of the 50 lowest normal modes.**

7. In **Fig 3**, “The black dotted lines represent the corresponding experimental resolution of the proteins.” Sorry, I don’t understand the description here. As the vertical axis is RMSD. I also didn’t quite understand the meaning of frame in the horizontal axis. Are they predicted intermediated conformations? Why did authors use ~ 70 frames for ADK but ~ 60 frames for RBP?

**Response of the Authors:** In **Fig 3**, the RMSD in the vertical axis describes the spatial difference of intermediate structures with respect to the final structure. The frame in the horizontal axis means consecutive intermediate structures, so the total number of frames indicate the number of iteration steps of our methods. In the original manuscript, we have mistakenly omitted an explanation of how we determined the number of iteration steps. The number of iterations was determined so that the simulated intermediate structures comprise a pathway at intervals of an RMSD value of about 0.1 Å. In this study, we set the number of iterations for the ADK (RBP) pathway to 71 (62) because the RMSD value between the two end-point conformations is 7.16 Å (6.25 Å). In the revised manuscript, we add the following sentences on Page 16.

The number of iteration steps was determined so that consecutive intermediate structures differ by a root-mean-square deviation (RMSD) of about 0.1 Å. Here, we set the number of iteration steps for the case of ADK (RBP) is set to 71 (62) because the RMSD value between the two end-point conformations is 7.16 Å (6.25 Å).

8. Page 22, line 425, a “than” should be added after “reliable”.

**Response of the Authors:** According to referee’s suggestion, in the revised manuscript, this typo is fixed.

9. The color bar used in the **Fig 6** is very difficult to differentiate, the rainbow color scheme might be better.

**Response of the Authors:** According to referee’s suggestion, we represented the pathway ensemble using the rainbow color scheme (**Fig R1**). The most important point thing in this simulation result is that the pathways tended to closely approach the NMP_C_ state as the number of modes decreased. When using more than 35 normal modes, the resulting pathways are relatively straight, leading to that the rainbow color scheme may disturb rather than enhance visual perception in **Fig R1B** because too many lines are overlapped in the narrow region (having the straight pathways). Therefore, we think that the original version of **Fig 6** (*i.e.*, red to blue color scheme) would be more suitable for describing this simulation result.

**Fig R1. The performance of the pathway ensemble of ADK.** The ICONGENI transition pathways that make up the pathway ensemble are colored according to the lowest normal modes (from 5 to 100) used in the simulation (rainbow color scheme). (A) Convergence of the pathway ensemble. The RMSD values of each path relative to a final state are measured. The black dotted line represents the corresponding experimental resolution of the proteins. (B) Projection of the pathway ensemble onto $\theta_{NMP}$–$\theta_{LID}$ space. The green points show the positions of experimental structures. 4AKE:A and 1AKE:A indicate the open and closed states of ADK, respectively. 1ZIN:A, 1ZIO:A, and 1ZIP:A (1DVR:A, and 1DVR:B) indicate experimental structures at the NMP_C_ state (the NMP_O_ state).

10. The superiority was not emphasized enough by the way of presenting/writing in this manuscript. More analyses and comparisons should be conducted.

**Response of the Authors:** According to referee’s suggestion, we performed a further quantitative analysis to evaluate these two methods. In the same context as analyzing the variations of the internal coordinates (*i.e.*, backbone bond length and bond angle), we calculated the potential energy of the intermediate conformations on the pathways. Because both simulation methods were carried out by using a coarse-grained modeling method, non-backbone atoms and O atom in the backbone from reference structures (4AKE:A and 1BA2:A for ADK and RBP, respectively) were grafted to all intermediates and the generated all atom models were energy minimized within CHARMM36m force field [1] to eliminate any steric clashes and inappropriate geometries. Next, we calculated the potential energies of all intermediates of the NGENI and ICONGENI pathways by using CHARMM36m force field to quantitatively evaluate how the corresponding transitions are stable. **Fig R2** shows the difference of the potential energy of each frame between the NGENI and ICONGENI pathways. From the results, we confirm that the ICONGENI pathways are generally more stable than the NGENI pathways. Given that the geometric errors are gradually accumulated when anharmonic transitions are explored by harmonic modes, their energy gap increases as the pathways progress. These simulation results imply that the mode shapes in internal coordinates are more suitable for exploring large-scale transitions than those in Cartesian coordinates. In the revised manuscript, we clarify this point by adding the following sentences on Page 21 and **Fig R2** as **Fig 6**, with the following captions.

In the same context as investigating the bond length and bond angle distributions, the potential energies of the intermediate conformations comprising the resulting pathways were calculated. Because both simulation methods were carried out by using a coarse-grained modeling method, non-backbone atoms and O atom in the backbone from reference structures (4AKE:A and 1BA2:A for ADK and RBP, respectively) were grafted to all intermediates and the generated all atom models were energy minimized within CHARMM36m force field for 500 steps of conjugate gradient [37] to eliminate any steric clashes and inappropriate geometries. Next, we calculated the potential energies of all intermediates of the NGENI and ICONGENI pathways by using CHARMM36m force field to quantitatively evaluate how the corresponding transitions are stable. **Fig 6** shows the difference of the potential energy of each frame between the NGENI and ICONGENI pathways. From the results, we confirmed that the ICONGENI pathways are generally more stable (*i.e.*, having lower potential energies) than the NGENI pathways. Furthermore, their energy gap increased as the pathways progress, which suggests that the qualitative difference between the pathways is increasingly noticeable in that the geometric errors are gradually accumulated when anharmonic transitions are explored by harmonic modes. Finally, these simulation results imply that the ICONGNIE pathways are more reliable than the NGENI pathways in terms of thermal and chemical stability.

**Fig 6. The difference between potential energies of ICONGENI and NGENI pathways for (A) ADK and (B) RBP.** The difference of the potential energy $\Delta U=U_{ICONGENI}-U_{NGENI}$. Before calculating the potential energies, all simulated intermediate structures were transformed into all atom models based on corresponding reference structures (4AKE:A (1BA2:A) for ADK (RBP)) and were energy minimized using CHARMM36m force field for 500 steps of conjugate gradient.

**Fig R2. The difference between the potential energies of the ICONGENI and NGENI pathways for (A) ADK and (B) RBP.** The difference of the potential energy $\Delta U=U_{ICONGENI}-U_{NGENI}$. Before calculating the potential energies, all simulated intermediate structures were transformed into all atom models based on corresponding reference structures (4AKE:A (1BA2:A) for ADK (RBP)) and were energy minimized using CHARMM36m force field for 500 steps.

Furthermore, to demonstrate that ICONGENI can explore multiple transition pathways, we performed extra simulations where the paths via the NMP_O_ state not detected in the pathway ensemble simulated by ICONGENI were explored. The strategy was to use the normal mode set, composed of lowest normal modes characterizing the dynamics of $\theta_{LID}$ rather than those of $\theta_{NMP}$, as the DOFs of the simulations. As a result, ICONGNEI successfully generated the transition pathways via the NMP­_O_ state (**Fig R3**), which implies that if information of metastable states of a protein is given, our technique can explore the transition pathways compatible to the given states. In the revised manuscript, we add following sentences to discuss this point.

However, this result does not mean ICONGENI always returns a single candidate (*i.e.*, paths via the NMP_C_ state) of the transition paths. If the normal mode set as the system DOFs is determined under certain conditions, ICONGENI could explore transition pathways via the NMP_O_ state, which demonstrate that ICONGENI can explore multiple transition pathways compatible to several metastable states if information of the states is given (See more details in **S1 Text** and **S1 Fig**).

Furthermore, this discussion is included in the supporting information **S1 Text**. **Fig R2** is also included as **S1 Fig** with following captions.

**S1 Fig. Projection of the ICONGENI pathways for ADK onto** $\boldsymbol{\theta}_{\boldsymbol{NMP}}$**–**$\boldsymbol{\theta}_{\boldsymbol{LID}}$ **space.** Six pathways in the vicinity of the NMP_O_ state are shown on $\theta_{NMP}$–$\theta_{LID}$ space. The pathway named “diff. $a$ degrees” means that it was explored by ICONGENI using the lowest normal modes that satisfy the condition: $\Delta\theta_{LID}- \Delta\theta_{NMP}>a$ (see details in **S1 Text**). The ADK crystal structures are taken as the references (indicated by green circles). 4AKE:A and 1AKE:A indicate the open and closed states of ADK, respectively. 1ZIN:A, 1ZIO:A, and 1ZIP:A (1DVR:A, and 1DVR:B) indicate experimental structures at the NMP_C_ state (the NMP_O_ state). The pathway ensemble explored by ICONGENI is also shown on $\theta_{NMP}$–$\theta_{LID}$ space for comparison (The image in **Fig 6B** is blurred).

**Fig R3. Projection of the ICONGENI pathways for ADK onto** $\boldsymbol{\theta}_{\boldsymbol{NMP}}$**–**$\boldsymbol{\theta}_{\boldsymbol{LID}}$ **space.** Six pathways in the vicinity of the NMP_O_ state are shown on $\theta_{NMP}$–$\theta_{LID}$ space. To explore the NMP_O_ state, the specific normal modes that characterize the dynamics of $\theta_{LID}$ are used to generate the transition pathways. To select the desired normal modes, the changes of $\theta_{NMP}$ and $\theta_{LID}$ in the $k^{th}$ normal mode ($\left( \Delta\theta_{NMP} \right)_{k}$ and $\left( \Delta\theta_{LID} \right)_{k}$) are measured by the following equation: $\left( \Delta\theta_{NMP(or LID)} \right)_{k}=\left| \theta_{NMP(or LID)}\left( \boldsymbol{X} \right)-\theta_{NMP(or LID)}\left( \boldsymbol{X}_{deform,k} \right) \right|$, where $\boldsymbol{X}_{i}$ is the position vectors of atom $i$ of the intermediate structure and $\left( \boldsymbol{X}_{deform,k} \right)_{i}\boldsymbol{=}\boldsymbol{X}_{i}+50\times\Delta\boldsymbol{r}_{i,k}$ ($\Delta\boldsymbol{r}_{i,k}$ is the normalized eigenvector of atom $i$ in the $k^{th}$ normal mode). The pathway named “diff. $a$ degrees” means that ICONGENI uses the normal modes that satisfy the condition: $\Delta\theta_{LID}- \Delta\theta_{NMP}>a$. The ADK crystal structures are taken as the references (indicated by green circles). 4AKE:A and 1AKE:A indicate the open and closed states of ADK, respectively. 1ZIN:A, 1ZIO:A, and 1ZIP:A (1DVR:A, and 1DVR:B) indicate experimental structures at the NMP_C_ state (the NMP_O_ state). The existing pathway ensemble depending on the number of lowest normal modes is also shown on $\theta_{NMP}$–$\theta_{LID}$ space for comparison (The image in **Fig 6B** is blurred).

11. Is the link of code available? If so, I suggest to provide the link in Abstract.

**Response of the Authors:** The link of code data is available. When submitting the manuscript, we already agreed to provide the link information. If it is officially published in PLOS ONE, the link will be open to the public according to the PLOS ONE journal policy.

**References**

1. Frezza E, Lavery R. Internal Coordinate Normal Mode Analysis: A Strategy to Predict Protein Conformational Transitions. J Phys Chem B. 2019; 123(6):1294–1301. https://doi.org/10.1021/acs.jpcb.8b11913 PMID: 30665293
2. Lopéz-Blanco JR, Garzón JI, Chacón P. iMod: multipurpose normal mode analysis in internal coordinates. Bioinformatics. 2011; 27(20):2843–2850. https://doi.org/10.1093/bioinformatics/btr497 PMID: 21873636
3. Kitao A, Hayward S, Gō N. Comparison of normal mode analyses on a small globular protein in dihedral angle space and Cartesian coordinate space. Biophys Chem. 1994; 52(2):107–114. https://doi.org/10.1016/0301-4622(94)00070-0 PMID: 17020826
4. Kim MK, Chirikjian GS, Jernigan RL. Elastic models of conformational transitions in macromolecules. J Mol Graph and Model. 2002; 21:151–160. https://doi.org/10.1016/S1093-3263(02)00143-2 PMID: 12398345
5. Chakraborty S, Venkatramani R, Rao BJ, Asgeirsson B, Dandekar AM. Protein structure quality assessment based on the distance profiles of consecutive backbone Cα atoms. F1000Research. 2013; 2:211. https://doi.org/10.12688/f1000research.2-211.v3 PMID: 24555103
6. Lu M, Poon B, Ma J. A new method for coarse-grained elastic normal-mode analysis. J Chem Theory Comput. 2006; 2(3):464–471. https://doi.org/10.1021/ct050307u PMID: 21760758
7. Huang J, Rauscher S, Nawrocki G, Ran T, Feig M, De Groot BL, et al. CHARMM36m: an improved force field for folded and intrinsically disordered proteins. Nat Methods. 2017; 14(1):71–73. https://doi.org/10.1038/nmeth.4067 PMID: 27819658

**Reviewer #3:** This manuscript describes a new methodology based on Normal Mode Analysis framework to decipher protein conformational transitions by quantitatively capturing often elusive intermediate conformational states. While the methodology has merit, I have several major comments that the authors must address to render the manuscript suitable for publication in PLOS ONE.

1. The authors describe in the Introduction (line 51-53) section that "MD simulation has some problems (like those of experimental methods)". This sentence is written rather vaguely because the context of comparing MD methodology to numerous experimental techniques has to be clearly laid out, which has not been done by the authors. The authors must provide context and provide clear contextual justification when they are comparing the technical details (which are distinct) in experimental techniques and computational techniques, including MD simulation.

**Response of the Authors:** In the original manuscript, what we originally wanted to convey in the sentence “MD simulation has some problems (like those of experimental methods)” was that MD method has some limitations in exploring large-scale transitions of proteins like any other experimental technique, not that MD method and experimental techniques have the same limitations. In the revised manuscript, the sentence was modified for better clarification as follows.

However, despite its successful applications in numerous studies, MD simulation has intrinsic limitations in exploring the large-scale conformational changes: the simulated systems easily get trapped in stable or metastable states and rarely cross high-energy barriers toward functional states, even on millisecond time scales.

2. The authors must elucidate (line 73-74) regarding the novelty of NMA obtained in Internal Coordinates (IC). How does the choice/assignment of IC affect the underlying analysis framework? The authors should comment on this.

**Response of the Authors:** In the original manuscript, we already discussed the strength and weakness of IC-NMA compared to CC-NMA. Briefly speaking, CC-NMA has been widely used in studying protein dynamics due to the inherent nature of Cartesian coordinates (CCs): high computational efficiency and intuitive expression of protein dynamics but has a critical weakness that the resulting mode shapes unavoidably have unrealistic distortions like bond length stretching and bond angle bending [1]. On the other hand, IC-NMA requires higher computational cost than does CC-NMA because it has extra calculations involved in transformation either from CCs to ICs or from ICs to CCs. However, IC-NMA can provide chemically relevant mode shapes preserving the secondary structure of proteins and extending the validity of the harmonic approximation required for the calculation in that internal coordinates (ICs) define molecular geometry using bond length, bond angle, and torsion angle terms [1–3]. In the revised manuscript, we clarify this point by adding the following sentences on Page 4.

In this study, we propose a new NMA-based pathway generation method called internal coordinate normal mode-guided elastic network interpolation (ICONGENI), an improved technique over the normal mode-guided elastic network interpolation (NGENI) [25]. The fundamental concept of both methods is to obtain intermediate conformations comprising a transition pathway by iteratively calculating displacement vectors to minimize error between the initial structure and the desired intermediates. The critical difference between the two techniques is in NMA calculation performed to represent the displacement vectors as linear combinations of the lowest normal mode shapes: NMA calculation is achieved in internal coordinates (IC-NMA) and Cartesian coordinates (CC-NMA) in ICONGENI and NGENI, respectively. CC-NMA has been widely used in studying protein dynamics due to the inherent nature of Cartesian coordinates (CCs): high computational efficiency and intuitive expression of protein dynamics but has a critical weakness that the resulting mode shapes unavoidably have unrealistic distortions like bond length stretching and bond angle bending [26]. On the other hand, IC-NMA has a distinctive advantage in describing the large-scale transitions. It has been well known that large-scale transitions of proteins are dominantly influenced by the variations in the torsion angles, not in the bond lengths and the bond angles that are nearly rigid. Internal coordinates (ICs) facilitate to separate the torsion angle terms from the others, so that IC-NMA can be performed in torsion angle space. This strategy enables it to produce chemically relevant mode shapes preventing the unrealistic distortions of bond lengths and bond angles and extending the validity of the harmonic approximation in calculation [26–28]. In terms of computational complexity, IC-NMA is less efficient than CC-NMA because it has extra calculations involved in transformation either from CCs to ICs or from ICs to CCs (see section 2.2 for further details), but this is not a critical point in that both methods can be performed at the personal computer level. In other words, IC-NMA is more suitable than CC-NMA for describing and exploring large-scale conformational changes of proteins, which provides an insight into the development of ICONGENI.

3. Line 111: the authors must clearly state what they mean by conformational energy and how it is assigned to a conformational state.

**Response of the Authors:** In the original manuscript, we used the word “conformational energy” in the same sense as “free energy”. By projecting the resulting pathways onto the interdomain angle space that indirectly indicate the free energy landscapes and comparing them with crystal structures, we tried to evaluate them in terms of free energy.

In the revised manuscript, we modified “conformational energy” to “free energy” to prevent confusion.

4. Line 116: it is needed to justify why the authors chose DOPE score to select the best models corresponding to each template? Were any other scoring functions used? If not, then the authors must clearly weigh in on the merit of using the DOPE score as a selection criteria of their models.

**Response of the Authors:** In our manuscript, we used Modeller v9.25s to perform homology or comparative modeling of ADK crystal structures [4]. Therefore, we also used the DOPE assessment method that is implemented in the Modeller program. In detail, DOPE (discrete optimized protein energy) is a statistical potential used in prediction of 3D structures of protein. In the previous work, the Sali’s group confirmed that DOPE is the best performing function to detect the native states and find the optimal non-native model and that its score and alpha carbon RMS error have a high correlation [5].

5. Lines 127-128: The authors should comment on why curvilinear coordinates do not have a more significant impact on the relative spatial orientation of intermediate states.

**Response of the Authors:** Proteins have molecular geometry that are determined by internal coordinate terms like bond length, bond angle, and torsion angle, which implies that the protein dynamics are represented as variations in bond lengths, in bond angles, and in torsion angles (*i.e.*, relative motions of atoms). So, the internal coordinate system is suitable to describe the protein dynamics, while either the Cartesian coordinate system or the curvilinear coordinate system (like the polar coordinate system) has an intrinsic limitation in describing the dynamics in that these systems fundamentally specify the absolute positions of atoms in 3D space.

6. The sentence in lines 153-154 should be re-written for clarity.

**Response of the Authors:** According to referee’s suggestion, we re-write the sentence in lines 153-154 as follows.

The main strategy is to calculate the functions with respect to CCs and then to transform them into those with respect to ICs.

7. The authors should comment on why considering bond lengths and bond angles to be fixed does not affect the framework. While it is an approximation in their model, more quantitative justification is needed.

**Response of the Authors:** Actually, the protein transitions are mainly caused by the variations in torsion angles while the bond lengths and bond angles are unlikely to contribute to the transitions [6]. Given that the internal coordinate system can separate the chemically relevant variables (*i.e.*, torsion angles) from the others (*i.e.*, bond lengths and bond angles), IC-NMA is usually performed in torsion angle space, which prevents the resulting mode shapes having unrealistic distortions of bond lengths and bond angles [1–3]. In the revised manuscript, we add the following sentences on Page 4 for better clarification.

It has been well known that large-scale transitions of proteins are dominantly influenced by the variations in the torsion angles, not in the bond lengths and the bond angles that are nearly rigid. Internal coordinates (ICs) facilitate to separate the torsion angle terms from the others, so that IC-NMA can be performed in torsion angle space. This strategy enables it to produce chemically relevant mode shapes preventing the unrealistic distortions of bond lengths and bond angles and extending the validity of the harmonic approximation in calculation [26–28].

8. In Figure 1, the authors should comment on the validity of considering the two domains, A and B, as rigid bodies, and not consider local/distal fluctuations.

**Response of the Authors:** For calculation for a torsion angle term, we need a simple assumption that a molecular system is composed of several rigid bodies. Depending on the target torsion angle term, the virtual rigid-body domains are changed, which is valid only within the corresponding calculation. In other words, this assumption does not mean that a molecular system is actually composed of specific rigid bodies.

9. How does comparing the two approaches (previously developed, NGENI) and the current model (ICONGENI) ensure that the pathways considered are the same? Even setting all physical quantities in the respective calculations to be the same, how is it inherently ensured that they sample the same pathway? The authors must comment on/clarify this.

**Response of the Authors:** Although both methods are performed with the same physical quantities, the results are qualitatively different. In the original manuscript, **Figs 4** and **5** showed that ICONGENI generates more reliable pathways than does NGENI in terms of chemical energy. Furthermore, we performed another quantitative analysis to highlight the superiority of ICONGENI. In the same context as analyzing the variations of the internal coordinates (*i.e.*, backbone bond length and bond angle), we calculated the potential energy of the intermediate conformations on the pathways. Because both simulation methods were carried out by using a coarse-grained modeling method, non-backbone atoms and O atom in the backbone from reference structures (4AKE:A and 1BA2:A for ADK and RBP, respectively) were grafted to all intermediates and the generated all atom models were energy minimized within CHARMM36m force field [1] to eliminate any steric clashes and inappropriate geometries. Next, we calculated the potential energies of all intermediates of the NGENI and ICONGENI pathways by using CHARMM36m force field to quantitatively evaluate how the corresponding transitions are stable. **Fig R1** shows the difference of the potential energy of each frame between the NGENI and ICONGENI pathways. From the results, we confirm that the ICONGENI pathways are generally more stable than the NGENI pathways. Given that the geometric errors are gradually accumulated when anharmonic transitions are explored by harmonic modes, their energy gap increases as the pathways progress. These simulation results imply that the mode shapes in internal coordinates are more suitable for exploring large-scale transitions than those in Cartesian coordinates. In the revised manuscript, we clarify this point by adding the following sentences on Page 21 and **Fig R1** as **Fig 6**, with the following captions.

In the same context as investigating the bond length and bond angle distributions, the potential energies of the intermediate conformations comprising the resulting pathways were calculated. Because both simulation methods were carried out by using a coarse-grained modeling method, non-backbone atoms and O atom in the backbone from reference structures (4AKE:A and 1BA2:A for ADK and RBP, respectively) were grafted to all intermediates and the generated all atom models were energy minimized within CHARMM36m force field for 500 steps of conjugate gradient [37] to eliminate any steric clashes and inappropriate geometries. Next, we calculated the potential energies of all intermediates of the NGENI and ICONGENI pathways by using CHARMM36m force field to quantitatively evaluate how the corresponding transitions are stable. **Fig 6** shows the difference of the potential energy of each frame between the NGENI and ICONGENI pathways. From the results, we confirmed that the ICONGENI pathways are generally more stable (*i.e.*, have lower potential energies) than the NGENI pathways. Furthermore, their energy gap increased as the pathways progress, which suggests that the qualitative difference between the pathways is increasingly noticeable in that the geometric errors are gradually accumulated when anharmonic transitions are explored by harmonic modes. Finally, these simulation results imply that the ICONGNIE pathways are more reliable than the NGENI pathways in terms of thermal and chemical stability.

**Fig 6.** The difference between potential energies of ICONGENI and NGENI pathways for (A) ADK and (B) RBP. The difference of the potential energy $\Delta U=U_{ICONGENI}-U_{NGENI}$. Before calculating the potential energies, all simulated intermediate structures were transformed into all atom models based on corresponding reference structures (4AKE:A (1BA2:A) for ADK (RBP)) and were energy minimized using CHARMM36m force field for 500 steps of conjugate gradient.

**Fig R1. The difference between the potential energies of the ICONGENI and NGENI pathways for (A) ADK and (B) RBP.** The difference of the potential energy $\Delta U=U_{ICONGENI}-U_{NGENI}$. Before calculating the potential energies, all simulated intermediate structures were transformed into all atom models based on corresponding reference structures (4AKE:A (1BA2:A) for ADK (RBP)) and were energy minimized using CHARMM36m force field for 500 steps.

10. Lines 410-411 : The authors should clearly state what experimental structures were chosen for their experimental check? This sentence is ambiguous.

**Response of the Authors:** In the original manuscript, we introduced the structural information of the experimental structures as follows.

4AKE:A and 1AKE:A defines the two end-point conformations of the transition. The crystal structures whose PDB codes are 1ZIN, 1ZIO, and 1ZIP [37] and those whose PDB code is 1DVR [38] approximate the NMP_C_ state and the NMP_O_ state, respectively.

Moreover, we add the following sentence for better clarification in the revised manuscript.

In addition, we used some experimental structures for cross-validation with our simulation results.

11. The authors should comment on how do they ensure that given two end point conformations, they are sampling the same pathway in multiple iterations. How will the framework apply if they are considering open/closed transition of a protein that has metastable intermediate states which may be sampled at certain physical conditions?

**Response of the Authors:** Fundamentally, ICONGENI generates a deterministic pathway between given end-point structures under a specific condition (*e.g.*, a set of the lowest normal modes used in simulation and the number of iteration steps). In the original manuscript, we tried to generate the multiple transition paths for ADK depending on the number of the lowest normal modes used in ICONGENI simulation (**Fig 6**). Although the transition mechanisms of ADK, well known in previous studies [2], are divided into two scenarios: the transition via the NMP-closing/LID-opening (NMP_C_) state and that via the NMP-opening/LID-closing (NMP_O_) state, the pathway ensemble generated by ICONGENI supports only the existence of the NMP_C_ state (**Fig 6**). This simulation implies that the flexibility of the interdomain between NMP and CORE is higher than that between LID and CORE, resulting from that the vibrational features describing variations in the NMP-CORE angle are arranged in the lowest normal mode.

However, it does not mean ICONGENI always returns a single candidate of transition paths. To demonstrate that ICONGENI can explore multiple transition pathways, we performed extra simulations where the paths via the NMP_O_ state not detected in the pathway ensemble simulated by ICONGENI were explored. The strategy was to use the normal mode set, composed of lowest normal modes characterizing the dynamics of $\theta_{LID}$ rather than those of $\theta_{NMP}$, as the DOFs of the simulations. As a result, ICONGNEI successfully generated the transition pathways via the NMP­_O_ state (**Fig R2**), which implies that if information of metastable states of a protein is given, our technique can explore the transition pathways compatible to the given states. In the revised manuscript, we add following sentences to discuss this point.

However, this result does not mean ICONGENI always returns a single candidate (*i.e.*, paths via the NMP_C_ state) of the transition paths. If the normal mode set as the system DOFs is determined under certain conditions, ICONGENI could explore transition pathways via the NMP_O_ state, which demonstrate that ICONGENI can explore multiple transition pathways compatible to several metastable states if information of the states is given (See more details in **S1 Text** and **S1 Fig**).

Furthermore, this discussion is included in the supporting information **S1 Text**. **Fig R2** is also included as **S1 Fig** with following captions.

**S1 Fig. Projection of the ICONGENI pathways for ADK onto** $\boldsymbol{\theta}_{\boldsymbol{NMP}}$**–**$\boldsymbol{\theta}_{\boldsymbol{LID}}$ **space.** Six pathways in the vicinity of the NMP_O_ state are shown on $\theta_{NMP}$–$\theta_{LID}$ space. The pathway named “diff. $a$ degrees” means that it was explored by ICONGENI using the lowest normal modes that satisfy the condition: $\Delta\theta_{LID}- \Delta\theta_{NMP}>a$ (see details in **S1 Text**). The ADK crystal structures are taken as the references (indicated by green circles). 4AKE:A and 1AKE:A indicate the open and closed states of ADK, respectively. 1ZIN:A, 1ZIO:A, and 1ZIP:A (1DVR:A, and 1DVR:B) indicate experimental structures at the NMP_C_ state (the NMP_O_ state). The pathway ensemble explored by ICONGENI is also shown on $\theta_{NMP}$–$\theta_{LID}$ space for comparison (The image in **Fig 6B** is blurred).

**Fig R2. Projection of the ICONGENI pathways for ADK onto** $\boldsymbol{\theta}_{\boldsymbol{NMP}}$**–**$\boldsymbol{\theta}_{\boldsymbol{LID}}$ **space.** Six pathways in the vicinity of the NMP_O_ state are shown on $\theta_{NMP}$–$\theta_{LID}$ space. To explore the NMP_O_ state, the specific normal modes that characterize the dynamics of $\theta_{LID}$ are used to generate the transition pathways. To select the desired normal modes, the changes of $\theta_{NMP}$ and $\theta_{LID}$ in the $k^{th}$ normal mode ($\left( \Delta\theta_{NMP} \right)_{k}$ and $\left( \Delta\theta_{LID} \right)_{k}$) are measured by the following equation: $\left( \Delta\theta_{NMP(or LID)} \right)_{k}=\left| \theta_{NMP(or LID)}\left( \boldsymbol{X} \right)-\theta_{NMP(or LID)}\left( \boldsymbol{X}_{deform,k} \right) \right|$, where $\boldsymbol{X}_{i}$ is the position vectors of atom $i$ of the intermediate structure and $\left( \boldsymbol{X}_{deform,k} \right)_{i}\boldsymbol{=}\boldsymbol{X}_{i}+50\times\Delta\boldsymbol{r}_{i,k}$ ($\Delta\boldsymbol{r}_{i,k}$ is the normalized eigenvector of atom $i$ in the $k^{th}$ normal mode). The pathway named “diff. $a$ degrees” means that ICONGENI uses the normal modes that satisfy the condition: $\Delta\theta_{LID}- \Delta\theta_{NMP}>a$. The ADK crystal structures are taken as the references (indicated by green circles). 4AKE:A and 1AKE:A indicate the open and closed states of ADK, respectively. 1ZIN:A, 1ZIO:A, and 1ZIP:A (1DVR:A, and 1DVR:B) indicate experimental structures at the NMP_C_ state (the NMP_O_ state). The existing pathway ensemble depending on the number of lowest normal modes is also shown on $\theta_{NMP}$–$\theta_{LID}$ space for comparison (The image in **Fig 6B** is blurred).

12. The Conclusion section seems to have a broad overlap with the Materials and Methods section, and as such it needs to be re-organized. The authors must attempt to provide a comprehensive evaluation of the merits and limitations of their method when summarizing their models and its applicability to different types of systems. In addition, the figures must be re-formatted as they are of rather poor resolution.

**Response of the Authors:** According to referee’s suggestion, in the revised manuscript, we re-organized the Conclusion section as follows. Moreover, all figures were re-formatted as the improved versions with 600 dpi.

Given that the lowest normal modes in ICs have chemically relevant dynamics (suitable for describing large-scale transitions) compared to those in Cartesian coordinates, this strategy enables the proposed method to explore reliable transition pathways in an efficient manner.

To evaluate the superiority of ICONGENI, we performed comparative studies of ICONGENI with our previous method based on Cartesian coordinates (CCs) (named NGENI). For two proteins: adenylate kinase (ADK) and ribose-binding protein (RBP), we predicted transition pathways through the two methods under the same conditions (using the 50 lowest normal modes as the system degrees of freedom). From the distribution data of bond lengths and bond angles of the resulting pathways, it was confirmed that the internal coordinates remained highly stable in the ICONGENI pathways compared to those in the NGENI pathways (**Figs 4** and **5**). Furthermore, we also calculated the potential energies of the simulated pathways and identified the energies of the ICONGNEI pathways were lower overall than those of the NGENI pathways (**Fig 6**). In conclusion, these results suggest that the normal modes in ICs are suitable for representing realistic dynamics of the proteins, by extension, that ICONGENI could explore more reliable transition pathways than NGENI in terms of thermal and chemical stability.

Although ICONGENI using the DOFs of the 50 or more lowest normal modes can provide a spatial understanding of conformational transitions, this approach is insufficient to explain the actual transition events on complex energy landscapes. To overcome this limitation, we generated a pathway ensemble for ADK through ICONGENI depending on a set of lowest normal modes. First, we confirmed that the more is the number of normal modes used in the simulation, the closer the initial structure is to the final one, which is not surprising because the number of normal modes directly indicates the DOFs to describe structural dynamics (**Fig 7A**). Next, we characterized the pathway ensemble by interdomain angles of ADK (*i.e.*, the NMP-CORE angle ($\theta_{NMP})$ and the LID-CORE angle ($\theta_{LID})$) and found that the deficient pathways (using less than 50 lowest normal modes) provided meaningful insights into the conformational transitions of ADK. When projecting the ensemble onto $\theta_{NMP}$–$\theta_{LID}$ space, the deficient pathways showed the conformational transitions toward a metastable intermediate state (NMP_C_) while the sufficient pathways (using more than 50 lowest normal modes) showed those directly to the final state (*i.e.*, the closed state) regarded as unrealistic deformations (**Fig 7B**). Therefore, it is concluded that ICONGENI can explore meaningful transition pathways in terms of free energy landscapes.

The key role of computational approaches in investigating conformational transitions of proteins is to predict the trajectories that are beyond experimental capabilities. Our technique outlined here can shed light on the transition mechanisms in an efficient manner using only information on experimentally observed end-point structures. Furthermore, the simulation results strongly depend on a set of low-frequency normal modes as the system DOFs, enabling the method to explore pathway ensembles based on dynamic characteristics of proteins. Given that these ensembles can provide low-energy paths, our technique has the potential to find good candidates of unknown intermediate states on complex energy landscapes.

**References**

1. Frezza E, Lavery R. Internal Coordinate Normal Mode Analysis: A Strategy to Predict Protein Conformational Transitions. J Phys Chem B. 2019; 123(6):1294–1301. https://doi.org/10.1021/acs.jpcb.8b11913 PMID: 30665293
2. Lopéz-Blanco JR, Garzón JI, Chacón P. iMod: multipurpose normal mode analysis in internal coordinates. Bioinformatics. 2011; 27(20):2843–2850. https://doi.org/10.1093/bioinformatics/btr497 PMID: 21873636
3. Kitao A, Hayward S, Gō N. Comparison of normal mode analyses on a small globular protein in dihedral angle space and Cartesian coordinate space. Biophys Chem. 1994; 52(2):107–114. https://doi.org/10.1016/0301-4622(94)00070-0 PMID: 17020826
4. Webb B, Sali A. Comparative Protein Structure Modeling Using Modeller. Curr Protoc Bioinformatics. 2016; 54:5.6.1–5.6.37. https://doi.org/10.1002/cpbi.3 PMID: 27322406
5. Shen MY, Sali A. Statistical potential for assessment and prediction of protein structures. Protein Sci. 2006; 15(11): 2507–2524. https://doi.org/10.1110/ps.062416606 PMID: 17075131
6. Engh RA, Huber R. Accurate bond and angle parameters for X-ray protein structure refinement. Acta Cryst. 1991; A47(4):392–400. https://doi.org/10.1107/S0108767391001071
7. Huang J, Rauscher S, Nawrocki G, Ran T, Feig M, De Groot BL, et al. CHARMM36m: an improved force field for folded and intrinsically disordered proteins. Nat Methods. 2017; 14(1):71–73. https://doi.org/10.1038/nmeth.4067 PMID: 27819658
8. Wang Y, Gan L, Wang E, Wang J. Exploring the dynamic functional landscape of adenylate kinase modulated by substrates. J Chem Theory Comput. 2013; 9(1):84–95. https://doi.org/10.1021/ct300720s PMID: 26589012
